# Prediction of Patient Drug Response via 3D Bioprinted Gastric Cancer Model Utilized Patient‐Derived Tissue Laden Tissue‐Specific Bioink

**DOI:** 10.1002/advs.202411769

**Published:** 2025-01-02

**Authors:** Yoo‐mi Choi, Deukchae Na, Goeun Yoon, Jisoo Kim, Seoyeon Min, Hee‐Gyeong Yi, Soo‐Jeong Cho, Jae Hee Cho, Charles Lee, Jinah Jang

**Affiliations:** ^1^ Center for 3D Organ Printing and Stem cells (COPS) Pohang University of Science and Technology (POSTECH) Pohang 37666 Republic of Korea; ^2^ Ewha Institute of Convergence Medicine Ewha Womans University Mokdong Hospital Seoul 07985 Republic of Korea; ^3^ Department of Mechanical Engineering Pohang University of Science and Technology (POSTECH) Pohang 37666 Republic of Korea; ^4^ School of Interdisciplinary Bioscience and Bioengineering Pohang University of Science and Technology (POSTECH) Pohang 37666 Republic of Korea; ^5^ Department of Rural and Biosystems Engineering Chonnam National University Gwangju 61186 Republic of Korea; ^6^ Department of Internal Medicine Liver Research Institute Seoul National University Hospital Seoul 03080 Republic of Korea; ^7^ Department of Internal Medicine Gangnam Severance Hospital Yonsei University College of Medicine Seoul 06273 Republic of Korea; ^8^ The Jackson Laboratory for Genomic Medicine Farmington CT 06032 USA; ^9^ Department of Convergence IT Engineering Pohang University of Science and Technology (POSTECH) Pohang 37666 Republic of Korea; ^10^ Institute for Convergence Research and Education in Advanced Technology Yonsei University Seoul 03722 Republic of Korea

**Keywords:** drug efficacy testing, gastric cancer patient‐derived xenograft, gastric tissue‐derived decellularized extracellular matrix, tumor tissue printing

## Abstract

Despite significant research progress, tumor heterogeneity remains elusive, and its complexity poses a barrier to anticancer drug discovery and cancer treatment. Response to the same drug varies across patients, and the timing of treatment is an important factor in determining prognosis. Therefore, development of patient‐specific preclinical models that can predict a patient's drug response within a short period is imperative. In this study, a printed gastric cancer (pGC) model is developed for preclinical chemotherapy using extrusion‐based 3D bioprinting technology and tissue‐specific bioinks containing patient‐derived tumor chunks. The pGC model retained the original tumor characteristics and enabled rapid drug evaluation within 2 weeks of its isolation from the patient. In fact, it is confirmed that the drug response‐related gene profile of pGC tissues co‐cultured with human gastric fibroblasts (hGaFibro) is similar to that of patient tissues. This suggested that the application of the pGC model can potentially overcome the challenges associated with accurate drug evaluation in preclinical models (e.g., patient‐derived xenografts) owing to the deficiency of stromal cells derived from the patient. Consequently, the pGC model manifested a remarkable similarity with patients in terms of response to chemotherapy and prognostic predictability. Hence, it is considered a promising preclinical tool for personalized and precise treatments.

## Graphical abstract

This study presents a 3D bioprinted gastric cancer (pGC) model using patient‐derived tumor tissues and g‐dECM bioinks for personalized drug evaluation. The pGC model retains tumor and stromal cell interactions, enabling accurate chemotherapy response prediction within 2 weeks. It may offer a promising platform for assessing drug sensitivity, improving patient‐specific treatment, and advancing immunotherapy and targeted therapy research.

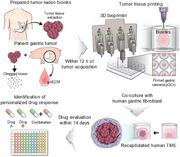

## 1 Introduction

Forecasting therapeutic outcomes stands as a crucial imperative in the realm of cancer management, aiming to mitigate adverse effects and enhance the effectiveness of anti‐cancer pharmacotherapy. Presently, the predominant approach in prognosticating the efficacy of anti‐cancer drugs revolves around gene panel testing predicated on next‐generation sequencing technologies.^[^ ^1^, ^2^, ^3^ ^]^ Despite its prevalence, gene panel testing confronts notable constraints. Particularly, a mere 10% of cancer patients harbor druggable mutations, even when employing comprehensive whole‐exome sequencing. Moreover, even in instances where optimal drugs are discerningly chosen, a substantive cohort of patients fails to accrue benefits from the prescribed pharmacological regimen. Additionally, the bulk of anti‐cancer drugs identified through gene panel testing fall within the molecularly targeted category, with the predictability of cytotoxic drugs' efficacies posing persistent challenges.^[^ ^4^, ^5^ ^]^ Besides, the selection of anticancer drugs for chemotherapy varies significantly across cancer types and individuals.^[^ ^6^ ^]^ Unfortunately, the current treatment paradigm often relies on limited clinical outcomes for drug selection with standardized protocols rather than being tailored to individual patient characteristics. Therefore, even in the context of allogeneic cancer, the response to drugs is inconsistent, leading to missed treatment opportunities or severe side effects since some patients exhibit positive responses while others do not.^[^ ^7^, ^8^ ^]^ Recently, endeavors to anticipate chemotherapeutic efficacy through *ex vivo* cultures derived from cancer patients are burgeoning. Various research cohorts have retrospectively elucidated associations between *ex vivo* drug sensitivity and clinical outcomes.^[^ ^9^, ^10^ ^]^ Besides, the patient‐derived tissue slice culture model has the advantage of preserving the TME, including histopathology, intercellular crosstalk, and various cell compositions, and has a shorter fabrication time than that required by patient‐derived xenograft (PDX) and patient‐derived organoids (PDOs) models.^[^ ^11^, ^12^ ^]^ Unfortunately, it can only be applied to malignant tumors and its lifespan is limited to 8 days on average.^[^ ^13^ ^]^ The primary source for *ex vivo* culture primarily hinges on surgically resected tumor tissue. However, patients grappling with recurrent or metastatic tumors typically circumvent surgical interventions, and chemotherapy is the sole recourse for those confronted with inoperable tumors. Therefore, imperative strides must be made toward predictive methodologies utilizing scant quantities of cancerous tissue procured from diagnostic materials such as endoscopic specimens, fine‐needle aspirates, and liquid biopsies.

To date, the numerous studies employing limited amounts of neoplastic tissue acquired from diagnostic substrates have various attempts to improve the precision of drug response prediction.^[^ ^14^, ^15^, ^16^, ^17^ ^]^ Currently, the PDX model is the prevailing preclinical model for evaluating drug efficacy and identifying new drugs.^[^ ^18^, ^19^, ^20^, ^21^ ^]^ PDX models, which are widely used in the field of cancer research, have significantly enhanced our understanding of cancer biology.^[^ ^22^, ^23^ ^]^ However, they have several disadvantages.^[^ ^24^ ^]^ First, the establishment of a comprehensive PDX cohort for in vivo drug screening requires a protracted period of 4–8 months. This time requirement significantly reduces the availability of screening results, especially considering that a significant proportion of patients with advanced or refractory cancers have a life expectancy of less than one year. Second, contingent on the tumor type, the rate of successful engraftment and growth of the impla6nted tumors exhibit substantial variability, ranging from 25% to 75%. Moreover, the maintenance of PDX mouse models is time‐consuming and expensive.^[^ ^25^ ^]^ Furthermore, the PDX model has a high risk of losing the stromal characteristics of cancer during the passage of patient‐derived cancer tissue into mice, leading to alterations in the properties of the tumor microenvironment (TME) and undergoing mouse‐specific tumor evolution.^[^ ^26^, ^27^ ^]^ These disadvantages render high‐throughput drug evaluation difficult and reduce drug accuracy.^[^ ^28^ ^]^ Meanwhile, there have been continuous trials using PDOs, which directly reflect a patient's genomic characteristics, for patient‐specific drug assessments in recent years.^[^ ^29^, ^30^, ^31^, ^32^, ^33^ ^]^ However, the PDOs model raises uncertainties about the preservation of the phenotype and characteristics of the original cancer, since it is primarily cultured without human stromal cells in a mouse sarcoma‐derived matrix known as Matrigel.^[^ ^34^, ^35^ ^]^ Therefore, the selection of appropriate culture materials is paramount for preserving the properties of cancer cells and faithfully recapitulating cell‐matrix interactions.^[^ ^36^, ^37^ ^]^ The extracellular matrix (ECM) in TME, endowed with biological activities, such as chemokines and signaling stimuli that are crucial for cancer cell survival and growth, has proven to be a valuable tool for modeling cancer‐specific characteristics, such as cell aggression, metastasis, and drug susceptibility.^[^ ^38^, ^39^, ^40^, ^41^, ^42^, ^43^, ^44^ ^]^ Recent studies have proposed constructing cancer models through the use of tissue‐specific decellularized ECM (dECM) components, obtained by decellularising target organs or tissues.^[^ ^45^, ^46^, ^47^, ^48^, ^49^, ^50^ ^]^ This matrix offers a complex molecular structure and 3D microenvironment that reflects the tissue.^[^ ^45^ ^]^ Thus, to ensure timely commencement of patient treatment, it is imperative to develop an in vitro cancer model that faithfully captures the genetic attributes of the patient, requires less construction time, and can be expanded in vitro, thereby facilitating highly efficacious drug estimation.

In this study, we proposed an in vitro 3D bioprinted cancer model, designed for the rapid evaluation of patient‐specific drug responses. We focused on gastric cancer (GC), a leading global cause of cancer‐related mortality, GC manifests considerable intertumoral heterogeneity, giving rise to discordant responses to anticancer therapies.^[^ ^51^ ^]^ By leveraging a dECM derived from porcine gastric tissue (g‐dECM), as developed in our previous study, we replicated the intricate microenvironment specific to GC.^[^ ^48^ ^]^ Our strategy was to mince patient‐derived cancer tissue without dissociating the cells and then immediately mixing it with g‐dECM to minimize the loss of cell‐to‐cell and cell‐to‐matrix interactions. Subsequently, by employing extrusion‐based 3D bioprinting technology and tissue‐specific bioinks containing patient‐derived cancer tissues, we produced hundreds of printed GC (pGC) specimens in a single printing session. In particular, the extrusion‐based 3D bioprinting approach enables several advantages; 1) Extrusion‐based techniques allow for printing at relatively low pressures and temperatures, which can maintain cell viability when using hydrogels containing living cells and reduce the risk of denaturation of key tissue‐derived protein components within the bioink. This approach is particularly useful when supporting the conservation of cell‐ ECM interactions during post‐printing in vitro culture.^[^ ^52^, ^53^ ^]^ 2) The user can easily adjust both pressure and flow rate, providing greater flexibility in handling bioinks with varying viscosities. In contrast, automated loading systems are generally optimized for lower‐viscosity bioinks, which can limit their application when working with high‐viscosity materials.^[^ ^54^, ^55^ ^]^ 3) The extrusion‐based bioprinting ensures precise control over the amount of bioink dispensed through the nozzle, thereby enhancing experimental reproducibility. This precise dispensing capability allows for consistent output, especially when conducting repetitive operations under the same pressure and flow rate, thus minimizing variability between experiments.^[^ ^56^, ^57^ ^]^ In addition, we co‐cultured pGC specimens with human gastric fibroblasts (hGaFibro), which play an important role in tumor development and drug resistance, to mimic the direct crosstalk and paracrine signaling between cancer cells and stromal cells in the TME. Finally, we assessed patient‐specific drug reactivity using the pGC model for chemotherapy with 5‐fluorouracil (5‐FU), docetaxel, and oxaliplatin, which are commonly used to treat patients with GC. Overall, we established a rapid, cost‐effective, scalable, and consistently reproducible approach for developing a pGC model that can be used for drug response assessment.

## 2 Results and Discussion

### 2.1 Tumor Tissue Printing using Patient‐Derived Tumor Laden Tissue‐Specific Bioinks and Evaluation of the Characteristics of printed gastric cancer (pGC) Model

**Figure 1:**
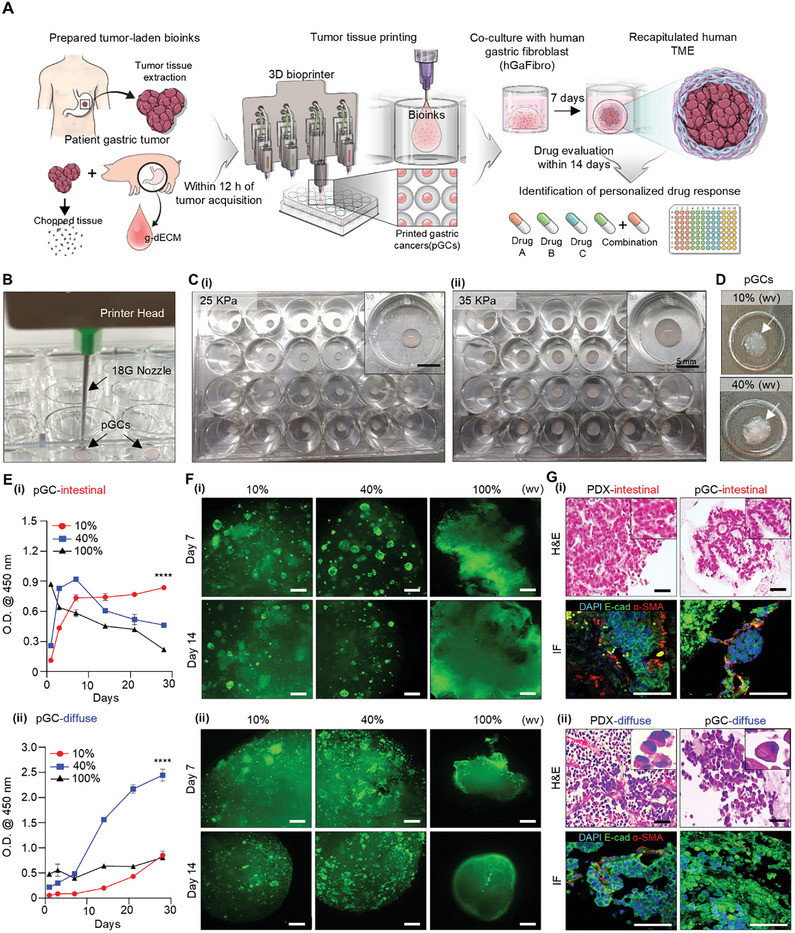
Tumor tissue printing using patient‐derived tumor‐laden tissue‐specific bioinks and evaluation of characteristics of pGC model. A) Schematic of the fabrication process for a 3D bioprinted model and its application in predicting patient drug responses. B) Production of pGC specimens and C) comparison of pGC sizes under different printing pressures of (i) 25 kPa and (ii) 35 kPa. D) Optical image of pGCs according to tumor tissue content ratio. E) Comparison of pGCs proliferation rate (i) pGC‐intestinal and (ii) pGC‐diffuse by tumor tissue content percentage for 21 days. The error bars represent the S.D. (n=3, ****p< 0.0001). F) Confirmation of culture suitability and the morphology of pGC‐intestinal and pGC‐diffuse tissues according to PDX tissue content using Calcein‐AM staining. Scale bars: 250 µm. G) Hematoxylin & Eosin (H&E) staining images and immunofluorescence staining images of E‐cadherin (green), α‐SMA (red), and DAPI (blue) in original PDX tissue and pGCs tissue at day 7, (i) intestinal type; (ii) diffuse type, Black scale bar: 50 µm; White scale bar: 100 µm. One‐way ANOVAs are used for the statistical analyses in (E).

We designed an in vitro model of GC tailored for clinical application, in order to expeditiously verify drug response and discern the efficacy of drugs for subsequent categorization as responsive or non‐responsive, with the aim of improving the efficiency of patient treatment within a clinically imperative timeframe through pre‐chemotherapeutic screening. We mixed biopsy‐obtained patient tissue in bioink within 12 h, produced tens to hundreds of specimens with a 3D bioprinter (i.e., 130 specimens produced with 1 g of patient tissue), and co‐cultured them with hGaFibro to recapitulate the cell‐matrix and cell‐cell interactions of the complex TME in humans. The pGC models could evaluate a patient's response to single or combination drug treatment within two weeks (**Figure** **1**A). To precisely manipulate cancer behavior, we used PDX tissue as a substitute for the patient to establish the printing and optimal culture conditions. We were able to quickly generate numerous replicas of specific dimensions by adjusting the printing parameters (e.g., nozzle size, pressure, and printing speed) (Figure 1B,C). Rapid contraction of the g‐dECM due to ECM remodeling by cells and cell‐matrix interactions may be a factor causing decreased cell proliferation.^[^ ^58^ ^]^ Therefore, a process of selecting an appropriate tissue concentration for bioink production would be necessary for stable cell proliferation. We investigated whether PDX‐derived cells within pGC tissue survived, proliferated well, and retained the inherent characteristics of the original cancer tissue. To determine the optimal in vitro culture conditions for GC tissue, cell proliferation rates were compared under three culture conditions. Various PDX tissues were encapsulated within the g‐dECM at concentrations of 10% or 40% weight per volume (w/v), with PDX tissues not encapsulated in the g‐dECM being represented by 100% (w/v) (Figure 1D). The prevalent morphological categories of GC predominantly follow Lauren (histological) classification. The latter delineates GC into distinct entities, namely intestinal (46‐54%), diffuse (32%), and indeterminate (15‐21%) types.^[^ ^59^, ^60^ ^]^ Intestinal type is marked by cohesive tumor cells that form glandular or intestinal like structures, resulting in an expansive growth pattern. In contrast, diffuse type features loosely cohesive tumor cells with minimal or absent glandular structures, leading to a growth pattern that diffusely infiltrates surrounding tissue.^[^ ^61^, ^62^ ^]^ As the two types show distinct phenotypes and clinicopathological features, we applied this classification to our research verifying the big difference between the two types. The intestinal and diffuse types of GC PDX tissues showed different cell proliferation and cell morphology in g‐dECM, respectively (Figure 1E,F). When pGC of the intestinal type (pGC‐intestinal) was generated under the condition of 10% (w/v), cell proliferation increased until day 7 of culture, and cell viability was maintained well from 7 to 28 days (Figure 1E(i)). In contrast, at 40% (w/v), rapid cell proliferation was observed until day 7 of culture, followed by a continuous decrease in cell viability from day 7 to 28. The 100% (w/v) tissue cultured without encapsulation in g‐dECM showed a sustained decrease in cell viability from day 1 to 28 of culture (Figure 1E(i)). Interestingly, results consistent with the cell proliferation curves were obtained within the calcein AM‐stained pGC intestinal tissues. Under the 10% (w/v) condition, similar number of aggregates existed in the 7th‐ and 14th‐day tissues, whereas under the 40% (w/v) condition, fewer aggregates were observed in the 14th‐day than in the 7th‐day tissue. In particular, the original PDX‐intestinal tissue pieces (100% (w/v)) showed intractability due to their very weak properties; therefore, they were easily broken and dissolved in the medium. This finding emphasized the crucial role of support materials (e.g., dECM) in the in vitro culture of cancer tissues with weak physical properties. In addition, the results showed that living cancer cells exist in aggregated forms (such as solid‐type organoids) in the pGC intestinal tissue. In particular, there was a clear difference in the number of aggregates in the culture depending on the amount of tissue initially included, and it affected the proliferation and culture stability of cancer cells (Figure 1F(i)). Consequently, the proliferation rate of cancer cells could be controlled by controlling the initial native tissue‐derived chuck volume.

The diffuse‐type pGC (pGC‐diffuse) showed the opposite tendency of cell proliferation compared to the pGC‐intestinal tissue (Figure 1E(ii)). The diffuse GC PDX tissue‐derived cells showed sustained cell proliferation for 28 d under both conditions of 10% (w/v) and 40% (w/v) g‐dECM, the cell proliferation rate being higher at 40% (w/v) than at 10% (w/v). The original diffuse‐type GC PDX tissue was harder than the intestinal‐type and did not dissolve in the medium; however, very little cell proliferation was observed here than in g‐dECM. In addition, more live cells were observed on day 7 than on day 14 by calcein AM staining, and aggregated cells were observed in pGC‐diffuse, just as in pGC‐intestinal tissue (Figure 1F(ii)). In particular, the pGC‐diffusion tissue aggregates were similar to grape clusters, whereas the pGC‐intestinal tissue aggregates had a bead‐like shape; therefore, the difference in aggregate morphology was distinct (Figure S1A, Supporting Information). This difference was thought to be due to the unique characteristics of cancer types or the levels of differentiation, and differences in the characteristics were more clearly confirmed by histological staining (Figure 1G). Hematoxylin and eosin (H&E) staining revealed the characteristic tubular structure of intestinal GC and the characteristic signet ring‐shaped cells of diffuse GC in both the original PDX tissue and the pGC model. In addition, we confirmed through immunofluorescence (IF) staining that cancer and stromal cells derived from each original PDX tissue were well preserved in the g‐dECM (Figure 1G; Figure S1B, Supporting Information). Additionally, we confirmed that there was no difference in the preservation, proliferation, and survival rates of patient‐derived cancer cells and stromal cells in g‐dECM than in Matrigel, which is widely used in in vitro cancer models (Figure S2, Supporting Information). Overall, our results demonstrated that mixing a small amount of patient cancer tissue with g‐dECM to generate a bioink, followed by the printing of numerous replicate pGC tissues, had no effect on cell proliferation and viability, and the original cancer characteristics were retained.

### 2.2 Assessment of Response of pGC Model to 5‐fluorouracil (5‐FU)‐ and Oxaliplatin‐Based Chemotherapy

**Figure 2:**
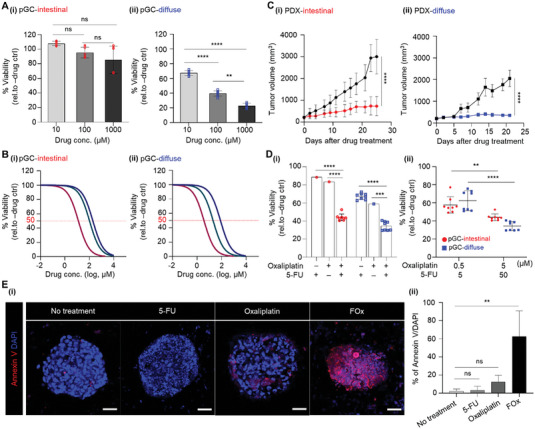
Assessment of responsiveness to 5‐FU and oxaliplatin‐based chemotherapy using pGC model. A) Evaluation of drug response in pGC tissues treated with various concentrations of 5‐FU. Data represent mean ± S.D. (n=3, ^**^ *p* < 0.01, ^****^ *p* < 0.0001, ns; not significant). B) Dose‐response curves after 3 days of treatment with 5‐FU, Docetaxel, and Oxaliplatin under different conditions for pGC‐intestinal and pGC‐diffuse. Representative viability curves were generated from the CCK‐8 assay. The IC_50_ values for each drug are described in Table 1. C) Assessment of the efficacy of the 5‐FU and Oxaliplatin combination regime in vivo and (D(i)) pGCs model. D(ii)) Two concentration combinations for 5‐FU and Oxaliplatin combination therapy. The error bars represent the S.D. (n=3, ^**^ *p* < 0.01, ^***^ *p* < 0.001 ^****^ *p* < 0.0001). E(i)) Immunofluorescence staining images of Annexun V (red) and DAPI (blue) in pGC‐intestinal with or without drug. Scale bar: 50 µm. E(ii)) Quantifications of fluorescence intensity of Annexin V normalized to DAPI. Data represent mean ± S.D. (n=3, ^**^ *p* < 0.01, ns; not significant). All measurement data was analysed using the ordinary two‐way ANOVA with Tukey's multiple comparisons test including *p*‐values.

Despite clear differences in the pathological characteristics and organotropism of the intestinal and diffuse types of GC, both entities are conventionally subjected to an analogous treatment strategy in routine clinical practice. The 5‐FU‐based therapy, including capecitabine + oxaliplatin (XELOX) and folic acid + 5‐FU + oxaliplatin (FOLFOX), is one of the first‐line chemotherapy regimens for advanced GC globally.^[^ ^63^ ^]^ Since the response to chemotherapy varies across patients, selection of an appropriate drug for the patient and initiating timely treatment are difficult. In this study, we investigated the drug reactivity of three anticancer agents (5‐FU, docetaxel, and oxaliplatin) in two pGC models with unique cancer characteristics from different patients. Additionally, pGC models were treated with a 5‐FU + oxaliplatin (FOx) regimen. When 5‐FU was treated with concentrations of 10, 100, and 1000 µM in pGC‐intestinal and pGC‐diffuse tissues, drug toxicity occurred in a concentration‐dependent manner (**Figure** **2**A). Moreover, in our pGC model, the resistance of pGC‐intestinal tissues to 5‐FU was higher than that of pGC‐diffuse tissues. Interestingly, the data contrasted with those of previous studies that had reported the resistance of diffuse GC to 5‐FU to be generally higher than that of intestinal GC. We attributed this result to the fact that our model represents patient characteristics. In addition, IC_50_ (the maximal inhibitory concentration) values for 5‐FU, docetaxel, and oxaliplatin were different between pGC‐intestinal and pGC‐diffuse tissues (*P* < 0.05, Figure 2B; **Table** **1**). The IC_50_ values in both the pGC models were significantly higher than those confirmed in the existing GC cell line model, and all three drugs showed higher resistance in the pGC‐intestinal model. In particular, oxaliplatin, a platinum‐based drug, exhibited the highest toxicity among the three drugs in both pGC‐intestinal and pGC‐diffuse tissues (Figure 2B; Table 1). Indeed, the patients who participated in the construction of GC PDX model were confirmed to respond to platinum‐based chemotherapy after GC resection.^[^ ^64^ ^]^ Not all patients with GC respond to 5‐FU or platinum‐based drugs, and the choice of chemotherapy drug is extremely important, since it affects patient prognosis. The current data provided crucial evidence that our pGC model sufficiently reflected the drug response in patients.

**Table 1:** IC_50_ values of three anticancer drugs on pGC models.

Drugs	^a)^ IC_50_ [µm]	
	pGC‐intestinal	pGC‐diffuse
5‐FU	772.4 ± 23.23	321.4 ± 18.41
Docetaxel	103.0 ± 9.00	20.19 ± 13.01
Oxaliplatin	15.12 ± 10.07	8.50 ± 8.85

^a)^ Data are the averages ± SD of two independent experiments conducted in triplicate (*p* < 0.05).

In particular, we focused on the fact that the FOx regimen exhibited tumor growth inhibition (TGI) effect in both intestinal and diffuse types in the PDX model (in vivo drug response results, Figure 2C). When treated with FOx regimen, the TGI effect was 92% and 78% in diffuse‐ and intestinal‐type GC PDX models, respectively, indicating that intestinal‐type PDX tissue was more resistant to 5‐FU‐ and oxaliplatin‐based chemotherapy.^[^ ^64^ ^]^ Indeed, when 5‐FU and oxaliplatin were used alone in pGC models, cytotoxicity was insignificant; however, when FOx regimen was used, cytotoxicity was more than 50%. In pGC‐intestinal tissue, treatment with 50 µm 5‐FU and 5 µm oxaliplatin showed 12% and 17% cell growth inhibition, respectively. In contrast, FOx regimen treatment represented a cell growth inhibitory effect of ≈62% (Figure 2D,E). Likewise, pGC‐diffuse tissue exhibited ≈73% cell growth inhibitory effect when treated with FOx regimen, compared to the ≈34% and 42% cell growth inhibitory effects, respectively, when treated with each drug alone at the same concentration (Figure 2D,E). Furthermore, the expression of Annexin V, an apoptosis marker, in the pGC‐intestinal tissue significantly increased upon combination treatment with 5‐FU and oxaliplatin (Figure 2F). In our pGC model, the higher resistance in pGC‐intestinal tissues than in pGC‐diffuse tissues, when treated with FOx regimen, was consistent with the drug response results of the PDX model (Figure 2C–E). In addition, we expanded our investigation to evaluate the synergistic effect of combination therapy with docetaxel and 5‐FU. Interestingly, no changes in toxicity were observed for the two concentrations of the docetaxel+5‐FU combination within pGC‐intestinal tissue, with toxicity levels ranging approximately from 11% to 13% and showing no statistical significance. However, in pGC‐diffuse tissues, a docetaxel to 5‐FU concentration ratio of 5:50 µm exhibited strong toxicity of ≈68% (Figure S3, Supporting Information). The results demonstrated the ability of the pGC model to adequately capture and reflect patient responses. Together, the drug reactivity assessment results indicated that the pGC model can be used to predict drug reactivity in patients. Furthermore, our pGC model strongly suggested that we can sensitively predict patient responses to drug treatment (either singly or in combination).

### 2.3 Production of the pGC Tissue‐Human Gastric Fibroblasts (hGaFibro) Co‐Culture Model and Its Molecular Biological Characterization with 5‐FU‐Based Chemotherapy

**Figure 3:**
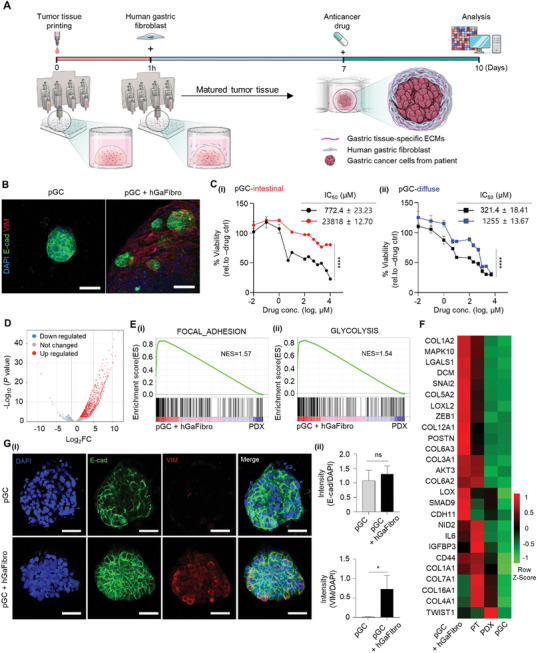
Identification of 5‐FU response‐related features in pGC with hGaFibro model. A) Schematic of the manufacturing process of pGC co‐culture with hGaFibro model for drug evaluation. B) Immunofluorescence staining images of E‐cadherin (green), Vimentin (red), and DAPI (blue) in pGC‐intestinal cultured in g‐dECM with or without hGaFibro. Scale bar: 100 µm. C) Dose‐response curves after 3 days of treatment with 5‐FU under various conditions of the presence and absence of hGaFibro in g‐dECM. Data represent mean ± S.D. (n=3, ^****^ *p* < 0.0001). D) Volcano plots represent the fold change and *P* value of up‐regulated (red) and down‐regulated (blue) differentially expressed genes (DEGs) on day 7 after pGC co‐culture with hGaFirbro models versus PDX models. x‐axis: Log_2_fold change (FC), Cutoff for Log_2_FC is 2. E) Gene set enrichment analysis (GSEA) of transcriptional profiles in pGC with hGaFibro models versus PDX models. E(i)) Enrichment plots of genes in hallmark FOCAL_ADHESION; E(ii)) hallmark_GLYCOLYSIS. (NES; normalized enrichment score, false discovery rate (FDR) *q‐*value <0.05). F) Heatmap showing the EMT‐related genes (PT; patient tissues). G(i)) Immunofluorescence staining images of E‐cadherin (green), Vimentin (red), and DAPI (blue) in pGC‐intestinal cultured in g‐dECM with or without hGaFibro. Scale bar: 50 µm. G(ii)) Quantifications of fluorescence intensity of E‐cadherin and Vimentin to normalized of DAPI. Data represent mean ± S.D. (n=3, ^*^ *p* < 0.05, ns; not significant). All measurement data was analyzed using the ordinary two‐way ANOVA with Tukey's multiple comparisons test including *p*‐values.

To propose a preclinical model that overcomes the limitations of the PDX or PDO model in terms of human stromal properties and the absence of stromal cells, we generated a co‐culture model with pGC tissue and primary hGaFibrothat reproduced the actual human GC microenvironment more closely (**Figure** **3**A). The pGC‐intestinal tissue co‐cultured with human hGaFibro showed a higher number of cancer cell aggregates, and cell‐to‐cell crosstalk between cancer cells and hGaFibro was expected through specific marker staining (Figure 3B; Figure S4, Supporting Information). In particular, the resistance to 5‐FU in both pGC‐intestinal and pGC‐diffuse tissues increased by ≈3‐ and fourfold, respectively, when co‐cultured with fibroblasts (Figure 3C). In general, diffuse‐type GC has a poorer prognosis than intestinal GC due to increased cancer aggressiveness and acquired multi‐drug resistance after chemotherapy. In contrast, 5‐FU resistance was higher in pGC intestinal tissues than in pGC diffuse tissues. In addition, the IC_50_ value was extremely high (>2 mM) in the co‐culture model with hGaFibro (Figure 3C). To investigate the cause, we analyzed the gene and protein expression profiles in pGC‐intestinal tissue with fibroblasts (pGC with hGaFibro; Figure 3D–G). We investigated the differentially expressed genes (DEGs) in the pGC‐intestinal tissue by RNA sequencing and identified 1015 up‐regulated genes in pGC with fibroblasts than in pGC without fibroblasts (Log_2_fold change >2, *P* < 0.05, Figure 3D). In addition, gene set enrichment analysis (GSEA, Figure 3E) based on DEGs in the pGC tissue revealed the enrichment of gene sets associated with cancer development and progression, such as growth, metabolism, and drug responsiveness, including “HALLMARK: Glycolysis” and “KEGG: Focal adhesion” in the pGC with hGaFibro model (Figure 3E). Since previous studies had shown that epithelial‐mesenchymal transition (EMT) is highly correlated with drug resistance in GC,^[^ ^65^, ^66^, ^67^ ^]^ we identified changes in the expression of EMT‐related genes and proteins (Figure 3F,G). Interestingly, the expression of genes representing EMT was significantly higher in pGC‐intestinal tissues with fibroblasts than in those without fibroblasts. In particular, the pGC tissue co‐cultured with human fibroblasts showed higher gene expression, similar to that in the patients″ cancer tissue, than in the PDX tissue (Figure 3F). To enhance clarity and support our findings, we conducted the principal component analysis (PCA) (Figure S4C, Supporting Information). It shows that the gene expression profile of pGC with hGaFibro tissue tends to be positioned closely with the PT tissue, while it remains distinct from the PDX or pGC tissue. This observation could be attributed to the possibility that co‐culture with fibroblasts enhances EMT‐related gene expression in a manner similar to what is observed in the patient's native cancer tissue. These findings suggest a promising trend, yet further validation through repeated experiments is warranted to strengthen the robustness of this observation. In addition, distinct expression of E‐cadherin, a cell junction and epithelial cell marker, was observed in the cancer cell aggregates of the pGC‐intestinal tissue, and the expression of vimentin, a representative EMT protein, was significantly increased upon co‐cultured with fibroblasts (Figure 3G). Changes in gene expression in PDX models associated with drug responses can lead to inaccurate drug evaluation. In contrast, pGC tissues co‐cultured with hGaFibro showed increased expression of genes involved in cancer cell metabolism, cell‐to‐cell interactions, and cell‐to‐ECM interactions. Furthermore, we confirmed that drug response‐related gene expression in pGC with hGaFibro was similar to those in patient tissues. The data were consistent with those of previous studies showing the interaction between stromal and cancer cells to play an important role in drug responsiveness, thereby highlighting the importance of crosstalk between stromal and cancer cells in drug evaluation.

### 2.4 Restoration of Key Drug Response‐Associated Pathways Found in Patient Tissues Upon Incorporation of hGaFibro into pGC Tissue

**Figure 4:**
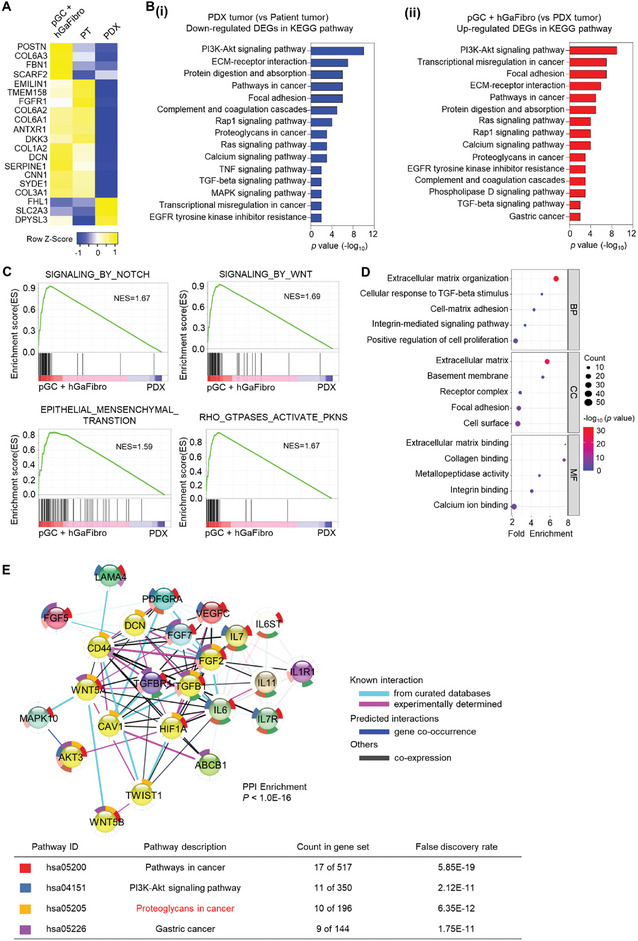
Comprehensive gene expression profiles associated with responsiveness to chemotherapy in pGC with hGaFibro models. A) Heatmap showing 20 drug resistance‐related genes (PT; patient tissues). B) Bar plots showing the most enriched KEGG pathways in the (i) down‐regulated genes in PDX tumor comparing to patient tumor and (ii) up‐regulated genes in pGC with hGaFibro comparing to PDX tumor. C) GSEA of the pGC with hGaFibro models versus PDX models. The pGC with hGaFibro models show Notch, Wnt, EMT, and Rho GTPase signaling pathways were enriched (NES; normalized enrichment score). D) Identification of significantly altered gene ontology (biological processes, cellular component, and molecular function) by GSEA (*q*‐value < 0.05) in the pGC with hGaFirbro models. E) Protein‐protein interaction (PPI) enrichment analysis utilizing the STRING network for the 469 DEGs exhibiting upregulation in the pGC with hGaFribro model. Enrichment *P‐*value are corrected for multiple testing using the method of Benjamini and Hochberg.

We identified the DEGs in patient tumor tissue, PDX tumor tissue, and pGC using the hGaFibro model to determine the effect of crosstalk between the printed cancer models and gastric fibroblasts on drug response. Interestingly, for the expression of 20 out of 32 genes associated with drug response in patients with GC,^[^ ^64^ ^]^ the similarity between patient tumor tissue‐pGC and the hGaFibro model was higher than that between patient tumor tissue‐PDX tumor tissue (**Figure** **4**A). Besides, the 2D PCA plot demonstrates a higher correlation between pGC+hGaFbiro tissue and PT tissue of drug response behavior (Figure S5, Supporting Information). This was an important evidence suggesting that our model, in which hGaFibro are co‐cultured on printed cancer tissue, can more closely predict drug responses in patients than the PDX model. Impressively, cancer proliferation, progression, and drug response‐related gene expression were reduced when PDX models were generated from patient cancer tissues, whereas they were restored in pGC tissues co‐cultured with hGaFibro (Figure 4B). In particular, the GSEA results of PDX tumor tissue and pGC in the hGaFibro model showed enrichment of Wnt‐, Notch‐, and EMT‐related genes in pGC (Figure 4C). Gene ontology (GO) analysis results based on 469 DEGs that were significantly upregulated in pGCs with the hGaFibro model than in PDX tumor tissue supported the enrichment of ECM remodeling and cell‐to‐ECM interactions in pGCs with the hGaFibro model (Figure 4F). Furthermore, protein‐protein interaction (PPI) networks of the up‐regulated DEGs in the pGC with hGaFibro model, compared to that in the PDX tumor, revealed the significant enrichment of “PI3K‐Akt signaling,” “Proteoglycan in cancer,” and “Gastric cancer” gene sets (Figure 4E). Proteoglycans, such as CD44, CAV1, HIF1A, and TGFB1, are known to be involved in metastasis and acquisition of drug resistance in GC.^[^ ^68^, ^69^, ^70^, ^71^, ^72^ ^]^ Thus, the results demonstrated that our pGC model improved the interaction between cancer cells and the ECM affecting drug response through co‐culture with fibroblasts. Therefore, we expected our model to predict patient drug responses more accurately than the existing drug evaluation models.

### 2.5 Investigation of the Response of the pGC‐Biopsy Tissue Model to 5‐FU and Oxaliplatin‐Based Chemotherapy

**Figure 5:**
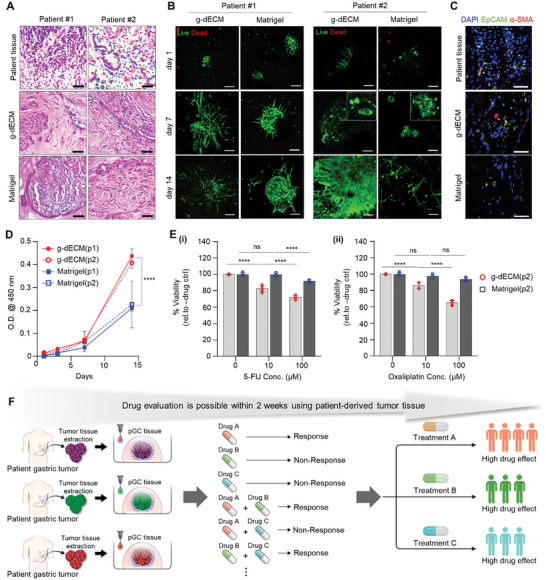
Investigation of responsiveness to 5‐FU and oxaliplatin‐based chemotherapy in a pGC‐biopsy tissue model. A) Hematoxylin & Eosin (H&E) staining of patient‐derived gastric tumor tissue. Scale bar: 50 µm B) Analysis of viability, culture suitability, and the morphology of pGC‐biopsy tissues in g‐dECM and Matrigel using live/dead staining. Scale bars: 200 µm. C) Immunofluorescence staining images of EpCAM (green), α‐SMA (red), and DAPI (blue) at day 7. Scale bars: 50 µm. D) Comparison of pGC‐biopsy tissue proliferation rates according to material type for 14 days. The error bars represent the S.D. (n=3, ^****^ *p* < 0.0001). E) Evaluation of drug response in pGC‐biopy tissue encapsulated in g‐dECM and Matrigel treated with various concentrations of (i) 5‐FU and (ii) oxaliplatin, respectively. Data represent mean ± S.D. (n=3, ^***^ *p* < 0.001, ^****^ *p* < 0.0001, ns; not significant). F) Summary of a model for rapid drug efficacy evaluation within 2 weeks using patient‐derived tumor tissue. All measurement data was analyzed using the ordinary two‐way ANOVA with Tukey's multiple comparisons test including *p*‐values.

A small amount of GC tissue obtained from the biopsy of a patient with GC was mixed with the g‐dECM and printed to produce a pGC‐biopsy tissue model. Cancer cells derived from patients are very difficult to culture in vitro, and establishing the optimal cultural conditions remains complex and challenging.^[^ ^73^ ^]^ We eliminated the complicated culture optimizing procedure and shortened the incubation time by encapsulating the patient's cancer tissue in g‐dECM. In the pGC‐biopsy tissue of Matrigel, cytotoxicity was not observed at drug concentrations of 10 µM and 100 µM when treated with 5‐FU. Conversely, in the pGC‐biopsy tissue of g‐dECM, cytotoxicity of 5‐FU was ≈18% at 10 µM and ≈28% at 100 µM (**Figure** **5**E(i)). Notably, response to oxaliplatin, a platinum‐based drug recognized for its heightened toxicity compared to 5‐FU, manifested strikingly different outcomes. Matrigel demonstrated little drug toxicity depending on concentration, whereas g‐dECM exhibited cytotoxicity of ≈14% at 10 µm and 35% at 100 µm, surpassing the toxicity observed with 5‐FU (Figure 5E(ii)).

Since the patients contributing to the development of the pGC tissue model only underwent surgical resection and radiation therapy without drug treatment, comparison of their response to 5‐FU and oxaliplatin was challenging. Nevertheless, the discernible contrast in drug reactivity between the two materials (Matrigel vs g‐dECM) for the same patient cells provided clear evidence of the substantial impact of cell‐ECM interactions in drug evaluation. Based on these findings, we asserted that a gastric tissue‐specific ECM grounded in cancer cell‐ECM interactions holds the potential for more sensitive drug response predictions compared to existing materials.

Meanwhile, we verified whether the pGC model developed in this study can be extended to other solid cancer models (e.g., pacreatic cacner, breast cancer, and melanoma, etc.). We encapsulated fine needle biopsy tissue from a pancreatic cancer patient into pancreatic dECM to produce bioink and generated a pancreatic cancer model under identical printing conditions (Figure S6A, Supporting Information). It was confirmed that pPC tissue was created with the same size as pGC tissue, which was ≈5 mm (Figure S6B, Supporting Information). In addition, compared to Matrigel, it was proved that patient‐derived cells were stably cultured in vitro for 2 weeks, and the simultaneous presence of cancer cells and stromal cells was observed (Figure S6C, Supporting Information). This is a meaningful result that demonstrates the potential of being able to test drugs within 2 weeks using a very small amount of biopsy tissue from pancreatic cancer patients. Therefore, to predict a patient's drug response accurately, composition of the matrix surrounding the cancer cells and presence of stromal cells is crucial. We anticipated that our printed cancer model would serve as a valuable tool for the expeditious and precise prediction of a patient's drug response (Figure 5F).

## 3 Conclusion

Our data provided proof‐of‐concept for the production of a patient‐specific in vitro cancer model for drug evaluation via 3D tissue bioprinting with tumor‐laden bioinks. The matrix surrounding the cancer cells in the TME contributes to cancer cell progression, metastasis, and sensitivity to chemotherapy.^[^ ^74^, ^75^, ^76^, ^77^ ^]^ Here, g‐dECM bioinks derived from porcine gastric tissue were used to support cell‐ECM interaction‐mediated cellular stimulation and signaling in the GC microenvironment. By directly generating printed cancer tissues using the g‐dECM bioink in multi‐well plates, the strategy enabled the production of in vitro cancer models that can effectively evaluate chemotherapy in a short time with minimal loss of patient characteristics (Figures 1 and 2). In particular, the pGC model not only validated the response to single‐drug treatment, but also evaluated the effect of combination treatment (Figure 2). The ability of our printed cancer model to reproduce patient‐derived cancer cells while retaining patient‐specific pathophysiological characteristics enabled an unprecedented evaluation of patient‐specific treatment responses.

The importance of cancer cell‐stromal cell crosstalk in anticancer drug efficacy and toxicity evaluation models has been continuously emphasized.^[^ ^78^ ^]^ However, numerous existing anticancer drug test models (e.g., cancer spheroids, PDO, and PDX models) still have limitations in their evaluation, due to the absence of stromal cells. Our printed cancer model reproduced the cancer cell‐matrix and cancer cell‐stromal cell crosstalk. The pGC tissue co‐cultured with stromal cells (e.g., fibroblasts and immune cells) showed changes in drug responses according to the cancer cell‐stromal cell crosstalk (Figure 3C; Figure S6, Supporting Information). Interestingly, the pGC model showed increased drug resistance when co‐cultured with fibroblasts; however, sensitive in reactivity was observed upon co‐culture with immune cells. In particular, our pGC model can be considered to contain patient‐derived immune cells present in the PDX tissue, as it focuses on minimizing the loss of patient‐derived cells. Strategically, we aimed to incorporate Jurkat cells with CD4+ helper T cell (T_h_) properties into the pGC model to enhance immune cells with reduced activation. Remarkably, the pGC‐intestinal+T_h_ model showed increased sensitivity to the 5‐FU, whereas the pGC‐diffuse+T_h_ model showed no difference in responsiveness to the 5‐FU (Figure S7, Supporting Information). T cells in the TME interact directly or indirectly with cancer cells, mainly through cytokine secretion, to activate other nearby immune cells (e.g., cancer‐associated macrophages, dendritic cells, and natural killer cells) and affect drug response.^[^ ^79^ ^]^ The observed heightened sensitivity to drug responses in pGC co‐cultured with helper T cells in this study can be attributed to the secretion of effector cytokines, notably interferon‐γ (IFN‐γ) and tumor necrosis factor‐α (TNF‐α), and this phenomenon is likely due to their direct cytotoxic impact on cancer cells within the TME.^[^ ^80^, ^81^ ^]^ Besides, several retrospective cohort studies have shown that the immune microenvironment score of patients with intestinal‐type GC is significantly higher than that of patients with diffuse‐type GC. In addition, a high immune cell score of GC tissue was significantly associated with prognosis only in intestinal‐type cancer, while in diffuse‐type GC, the ICS level was not associated with prognosis.^[^ ^82^, ^83^ ^]^ In conclusion, the more sensitive drug response in the pGC‐intestinal+T_h_ model indicates that GC cell‐immune cell interactions may differ depending on the intestinal and diffuse types of GC, which is consistent with the results of clinical studies showing that the prognosis of patients with intestinal‐type GC with an activated immune microenvironment is better than that of patients with diffuse‐type GC.^[^ ^84^, ^85^, ^86^ ^]^ In contrast, cancer cells can be directly stimulated by the cytokines and chemokines secreted by fibroblasts to activate pathways and signals related to drug resistance.^[^ ^87^ ^]^ To investigate the effect of recapitulating cancer‐fibroblast crosstalk within pGCs on drug responses, we conducted RNA sequencing on pGC tissue monocultures and pGC tissues co‐cultured with gastric fibroblasts (Figure 3D–F). Gene expression results suggested that the addition of gastric fibroblasts enhances the recapitulation of pathways (e.g., ECM‐receptor interactions, focal adhesion, and EMT‐related pathways) associated with ECM‐mediated cancer‐fibroblast crosstalk found in patient tumor tissues (Figure 3D–F). Surprisingly, the expression of genes related to cancer proliferation, progression, and drug response, which was reduced when generating the PDX model in the patient's cancer tissue, was mostly restored in pGC tissue co‐cultured with hGaFibro (Figure 4A,B). In particular, the functional enrichment related to “cell‐matrix adhesion (GO:0007160)”, “integrin binding (GO:0005178)”, and “integrin‐mediated signaling pathway (GO:0007229)” identified in the GO analysis of our printed cancer model showed clear evidence for enhanced cancer cell‐ECM interactions (Figure 4D).

In a pGC model containing biopsy tissues from patients, the trends of drug response to 5‐FU and oxaliplatin were different in Matrigel and g‐dECM, respectively (Figure 5E,F). Our results supported the existing claim that the effects of cell‐ECM interactions should be fully considered in models to evaluate drug efficacy or toxicity. The g‐dECM used in the pGC model not only contained various ECMs, such as gastric tissue‐derived proteoglycan and glycosaminoglycan, which are lacking in Matrigel, but also included cytokines produced and secreted by stromal cells, such as immune cells, fibroblasts, and endothelial cells (Figure S8, Supporting Information). Therefore, g‐dECM can support signal transduction and cellular stimulation of cancer cell initiation, development, and drug response. The pGC model generated with 3D tissue printing technology enabled the evaluation of patient‐specific drug responses within a short period of time without the establishment of demanding culture conditions.

In conclusion, the 3D bioprintied patient‐specific drug response prediction model developed in this study can be used to evaluate the drug sensitivity of patients before chemotherapy using actual cancer tissue and to identify an appropriate drug for each patient. Future studies may challenge the rapid evaluation of immunotherapy by creating a pGC model using a patient's biopsy or surgical tissue without using an animal model or co‐culturing immune cells obtained from the patient. Above all, our model can contribute to reducing reckless drug use in non‐responders, further improving patient prognosis and minimizing side effects. In addition, we expect it to be used for the discovery of new drugs, validation of the efficacy of drug combinations, and the development of personalized targeted therapies.

## 4 Experimental Section

### Patient Sample Collection

This study received approval from the Institutional Review Board at both Seoul National University Hospital (Approval No. H‐2302‐036‐1402) and Gangnam Severance Hospital (Approval No. 3‐2020‐0238), adhering to the Declaration of Helsinki. Patient samples were collected with informed consent at the respective institutions. Tumor tissue samples were obtained from patients who underwent gastrectomy at Seoul National University Hospital and Gangnam Severance Hospital in 2021. Individuals who had received preoperative chemotherapy or chemoradiation therapy were excluded from the study. Immediately following collection the tumor tissue, was placed in RPMI 1640 medium (Gibco) supplemented with 1% penicillin/streptomycin (Gibco) to maintain the optimal condition of the tumor tissue before tumor tissue printing.

### Generation of Patient‐Derived Xenograft (PDX) Models

Mice were maintained in compliance with the guidelines of the Institutional Animal Care and Use Committee of Seoul National University Hospital (Approval No. C‐1402‐054‐555). The procedure for generating PDX models was based on a previous study.^[^ ^64^ ^]^ In brief, surgically resected tumor tissues were minced into pieces ≈2 mm in size and implanted subcutaneously into the flanks of 6‐week‐old female NOD/SCID/IL‐2γ‐receptor null (NSG) mice (The Jackson Laboratory). Tumor volumes and body weights were monitored once or twice weekly. Tumor volume was calculated using the formula (length × width^2^)/2. Successful engraftment was considered as tumor formation at the implantation site exceeding 500 mm^3^. Upon achieving successful engraftment, mice were sacrificed, and the tumor tissues were harvested and preserved.

### In Vivo Pharmacological Studies

In PDX mice with subcutaneous engraftments of established tumors, drug treatments were initiated once the tumors reached ≈200 mm^3^ in size. The mice were randomly assigned to either a control group or a treatment group receiving 5‐FU and oxaliplatin, with five mice per group. The treatment regimen consisted of 5‐FU (Selleckchem, 5 mg kg^−1^, weekly) and oxaliplatin (Selleckchem, 50 mg kg^−1^, weekly), both administered via intraperitoneal injection in saline for 21 days. Tumor volumes were monitored three times per week and calculated using the formula (length × width^2^)/2. To assess treatment response, two criteria were used based on relative tumor volume changes between drug‐ and vehicle‐treated groups: significant inhibition of tumor growth by the drug (two‐way ANOVA, *P* < 0.0001 for the responder group, *P* > 0.05 for the non‐responder group). TGI is calculated via the following Equation (1):

(1) $$\mathrm{TGI}\;\left(\%\right)=\frac{TV_{\mathrm{vehicle}}-\;TV_{\mathrm{treatment}}}{TV_{\mathrm{vehicle}}-\;TV_{\mathrm{initial}}}\;X100$$

where TV_vehicle_ represents the tumor volume for vehicle‐treated animals at a designated endpoint, TV_initial_ is the initial tumor volume at the beginning of treatment, and TV_treatment_ is the tumor volume for the drug‐treated groups at the same endpoint. Samples not meeting these criteria were classified as the intermediate group. ANOVA testing was conducted using SPSS software, version 22 (IBM Corp., Version 22.0).

### Preparation of Gastric Decellularized Extracellular Matrix (g‐dECM)hGa Bioinks

The g‐dECM bioink was prepared following a previously described protocol.^[^ ^48^ ^]^ In brief, g‐dECM powder was dissolved in a 0.5 M acetic acid solution (#1.00063.2511, Merck Millipore, USA) containing 10% (w/w) pepsin (#7125, Sigma–Aldrich, USA) relative to the dECM weight. The mixture was stirred at 700 rpm for 120 h at 25 °C. To eliminate particulate impurities, the digested solution was filtered through a 70 µm cell strainer (#93040, SPL Life Sciences, Republic of Korea). The resulting dECM solution was then neutralized with 10 N sodium hydroxide (NaOH, #S2018‐1L, Biosesang, Republic of Korea) while keeping the temperature below 10 °C and stored at 4 °C.

### Tumor Tissue Priming and Generation of pGC Specimens

A microextrusion‐based 3D bioprinting system (3DXPrinter, T&R Biofab, South Korea) was used to generate pGC models. Tumor‐containing bioink was prepared by chopping the original tumor and encapsulating it in g‐dECM. The pGC specimens were fabricated in untreated 24‐well plates by extrusion through an 18G nozzle (DPN‐18G‐2, Musashi Engineering, Japan) using 35 kPa pneumatic pressure. The pGC model was cultured in a 37 °C incubator.

### Histological Analysis

The PDX tissue and g‐dECM were fixed in 4% paraformaldehyde (PFA, #CBPF‐9004, Chembio, USA) followed by dehydration, optical coherence tomography (OCT) embedding, sectioning, and staining using a Hematoxylin and Eosin (H&E) staining kit (ab245880, Abcam, USA) according to the manufacturer's protocol.

### The pGCs and Primary hGaFibro Culture

Human gastric fibroblasts (hGaFibro, # CC‐7231, Lonza, Switzerland) were cultured in RPMI‐1640 (#11875‐093, Gibco) supplemented with 10% fetal bovine serum (FBS, #TMS‐013‐BKR, Sigma‐Aldrich) and 1% penicillin/streptomycin (#sv30010, HyClone, USA). The hGaFibro were procured from P3, sub‐cultured, and used until P8. All cells were incubated at 37 °C in 5% CO_2_ and cultured all cells were detached from the culture dish using 0.05% Trypsin–EDTA (#25200‐056, HyClone). Cells were then resuspended in g‐dECM hydrogels or media at the concentrations required for each experimental condition.

### Live/Dead Assay

The viability of pGCs and hGaFibro were evaluated using a LIVE/DEAD Viability/Cytotoxicity Kit (#L3224, Thermo Fisher Scientific, USA). A staining solution was prepared by diluting 2 µL of calcein‐AM and 2 µL of ethidium homodimer‐1 in 1 mL of 1 × DPBS. A volume of 400 µL of this solution was added to each well of a 24‐well plate containing the samples, followed by incubation at 37 °C for 30 min. Fluorescent signals were captured using a confocal laser scanning microscope (Nikon Ti Eclipse, Nikon, Japan) and analyzed with NIS‐Elements Advanced Research software.

### Cell Counting Kit‐8 (CCK‐8)d Assay

The proliferation rates of pGCs were assessed using the Cell Counting Kit‐8 (CCK‐8) assay (#CK04‐13, Dojindo Molecular Technologies, Japan) with a microplate spectrophotometer (Multiskan Go, Thermo Fisher Scientific). The CCK‐8 solution was diluted tenfold in RPMI medium, and 500 µL of the solution was added to each well containing samples. The plates were then incubated at 37 °C for 4 h. Following incubation, 100 µL of the solution from each well was transferred to a 96‐well plate for triplicate analysis. Absorbance was measured at 450 nm to quantify cell proliferation.

### Cytokine Arrays

The proteins of g‐dECM and Matrigel were extracted by RIPA buffer (#RC2002‐050‐00, Biosesang, Republic of Korea) and then quantified by Pierce BCA Protein Assay Kits (#23225, Thermo Fisher Scientific, USA). The protein concentration was adjusted to 10 mg mL^−1^. Residual cytokine was measured by Porcine cytokine C1 (#AAP‐CYT‐1, RayBiotech, Inc., USA) according to the manufacturer's protocol.

### Drug Responsiveness Testing

The pGC were cultured for 7 days in a 24‐well plate and then treated with 10 different concentrations of 5‐FU (#S1209, Selleckchem, USA), Oxaliplatin (#S1224, Selleckchem), and Docetaxel (#S1148, Selleckchem, USA), with DMSO (#D8418, Sigma–Aldrich, USA) serving as a control. After 3 days of drug treatment, the pGCs were transferred to a new 24‐well plate, and 500 µL of CCK‐8 solution was added to each well to assess cell viability. Following incubation at 37 °C for 4 h, 100 µL of the solution was transferred to a 96‐well plate for triplicate measurements. Absorbance was measured at 450 nm, and IC_50_ values were calculated using GraphPad Prism version 8.4.3.

### Immunofluorescence (IF) Staining

For IF staining, pGCs were fixed in 4% PFA for 20 min at 25 °C, washed thrice in PBS. The resulting pGCs samples were permeabilized with 0.5% TritonX‐100 (#T1020, Biosesang)/PBS for 1 h, and blocked with 10% normal goat serum in 0.05% Triton X‐100/PBS for 1 h at 25 °C. The samples were then incubated with primary antibodies for 16 h at 4 °C. To delineate the cellular organization of pGCs, the following specific markers were used. The anti‐E cadherin (E‐cad; 1:200 dilution; #ab7753, Abcam), anti‐Pan cytokeratin (PanCK; 1:200 dilution; #ab7753, Abcam), anti‐Cytokeratin 19 (CK19; 1:200 dilution; #ab52625, Abcam), and anti‐Epithelial cell adhesion molecule (EpCAM; 1:200 dilution; #ab71916, Abcam) were used to stain pGCs. The anti‐Vimentin (VIM; 1:200 dilution; #ab8978, Abcam) and anti‐alpha smooth muscle (α‐SMA; 1:200 dilution; #ab7817, Abcam) were used to stain fibroblast. Lastly, anti‐Annexin V (1:200 dilution; #ab54775, Abcam) were used to confirm cytotoxicity of anticancer drug. After three PBS washes, the samples were incubated with secondary antibodies, Alexa Fluor 488 (1:200, #A‐11008, Invitrogen) and Alexa Fluor 594 (1:200, #A‐11005, Invitrogen), for 1 h at 25 °C. Following another set of three PBS washes, samples were mounted with a DAPI‐containing mounting medium (#H‐1500, VECTOR LAB, USA) to stain the nuclei.

Fluorescent imaging was conducted using a confocal laser scanning microscope (Nikon Ti Eclipse, Nikon, Japan), and images were processed and analyzed with NIS‐Elements Advanced Research software.

### TotalOmics Transcriptome

Libraries for 151 bp paired‐end sequencing were prepared using the TruSeq Stranded mRNA Sample Preparation Kit (Illumina, CA, USA). Specifically, mRNA was isolated and fragmented from 1 µg of total RNA using oligo(dT) magnetic beads. The fragmented mRNA was used to synthesize single‐stranded cDNA via random hexamer priming, which then served as a template for second‐strand synthesis to produce double‐stranded cDNA. After a series of steps including end repair, A‐tailing, and adapter ligation, the cDNA libraries were amplified using PCR. The quality of these libraries was assessed with the Agilent 2100 BioAnalyzer (Agilent, CA, USA), and quantification was performed using the KAPA Library Quantification Kit (Kapa Biosystems, MA, USA) according to the manufacturer's protocol. Sequencing was carried out on the Illumina NovaSeq 6000 system (Illumina, CA, USA) using paired‐end (2 × 151 bp) sequencing.

Adapter sequences and low‐quality ends (Phred score < 20) were trimmed and reads shorter than 50 bp were removed using Cutadapt v2.8.^[^ ^88^ ^]^ Filtered reads were aligned to the species‐specific reference genome using the STAR aligner v2.7.1a^[^ ^89^ ^]^ with ENCODE standard options and the “‐quantMode TranscriptomeSAM” option for estimating transcriptome expression levels.

Gene expression levels were estimated using RSEM v1.3.1,^[^ ^90^ ^]^ taking into account the strand specificity of the library protocol using the –strandedness option. The “–estimate‐rspd” option was also used to enhance measurement accuracy, with other parameters set to their default values. For normalization across samples, FPKM (Fragments Per Kilobase Million) and TPM (Transcripts Per Million) values were computed.

Using the read counts obtained in the previous step, differential expression genes (DEGs) were identified with the R package TCC v1.26.0,^[^ ^91^ ^]^ which employs robust normalization strategies for tag count comparisons. Normalization factors were calculated using an iterative DESeq2^[^ ^92^ ^]^/edgeR^[^ ^93^ ^]^ approach. The *q*‐value, an adjusted *p*‐value to correct for multiple testing, was calculated using the p.adjust function in R, with DEGs defined as those with q‐values less than 0.05.^[^ ^94^ ^]^

To functionally annotate DEGs, Gene Ontology (GO) analysis was performed using the R package GOseq^[^ ^95^ ^]^ based on the Wallenius non‐central hypergeometric distribution.^[^ ^96^ ^]^ GO terms are categorized into Biological Process (BP), Cellular Component (CC), and Molecular Function (MF). Genes with a *p*‐value < 0.05 in the GO trend test were considered statistically significant.

### Statistical Analysis

Data are presented as mean ± standard deviation (SD) based on three replicate samples. Statistical analyses were conducted using GraphPad Prism version 8.4.3 (GraphPad Software, USA). Group comparisons were made using multiple t‐tests (Holm‐Sidak method) or One‐way ANOVA and Two‐way ANOVA with Tukey's post‐hoc tests for multiple comparisons. Statistical significance is represented as follows: *p < 0.05, **p < 0.01, ***p < 0.001, and ****p < 0.0001.

## Conflict of Interest

The authors declare no conflict of interest.

## Supporting information



Supporting Information

## Data Availability

The data that supports the findings of this study are available in the supplementary material of this article
