# Supplementary material for: Prediction of Patient Drug Response via 3D Bioprinted Gastric Cancer Model Utilized Patient‐Derived Tissue Laden Tissue‐Specific Bioink
Source: Adv Sci (Weinh). 2025 Jan 2;12(10):2411769. doi: 10.1002/advs.202411769 (PMC11905052; doi:10.1002/advs.202411769)
Supplement: Supplementary file 1 — Supporting Information [file ADVS-12-2411769-s001.pdf]

ADVANCED SCIENCE
Open Access

Supporting Information

for *Adv. Sci.*, DOI 10.1002/advs.202411769

Prediction of Patient Drug Response via 3D Bioprinted Gastric Cancer Model Utilized Patient-Derived Tissue Laden Tissue-Specific Bioink

Yoo-mi Choi, Deukchae Na, Goeun Yoon, Jisoo Kim, Seoyeon Min, Hee-Gyeong Yi, Soo-Jeong Cho, Jae Hee Cho, Charles Lee and Jinah Jang**

Figure S1.

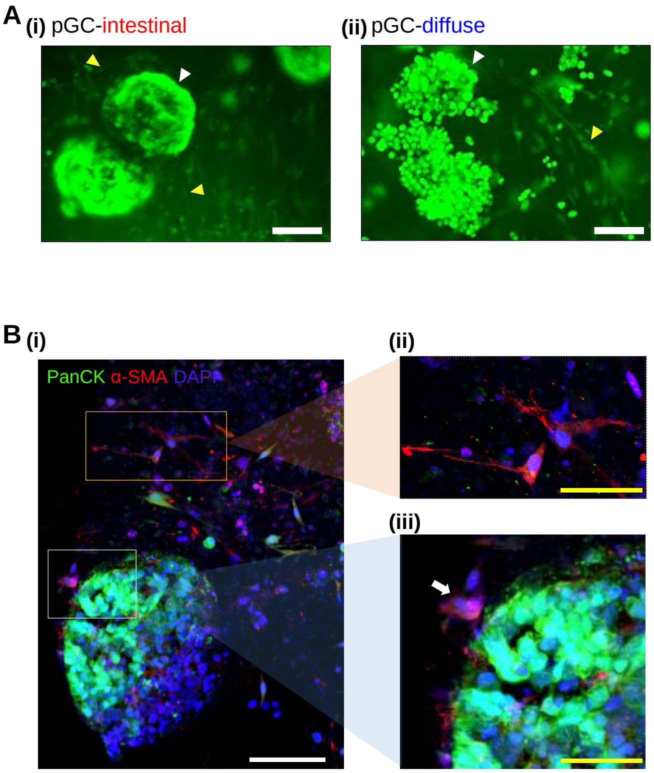

Figure S1. Observation of original tumor-derived cancer cells and stromal cells. (A) Comparison of the morphology of pGC-intestinal versus pGC-diffuse tissues using Calcein-AM staining at day 7. White arrow: cancer cells; Yellow arrow: stromal cells; Scale bars: 250 µm. (B) Immunofluorescence staining images of Pan cytokeratin (PanCK, green), α-SMA (red), and DAPI (blue) in pGC-intestinal tissues cultured in g-dECM at day 7. White scale bar: 100 µm; Yellow scale bar: 50 µm

Figure S2.

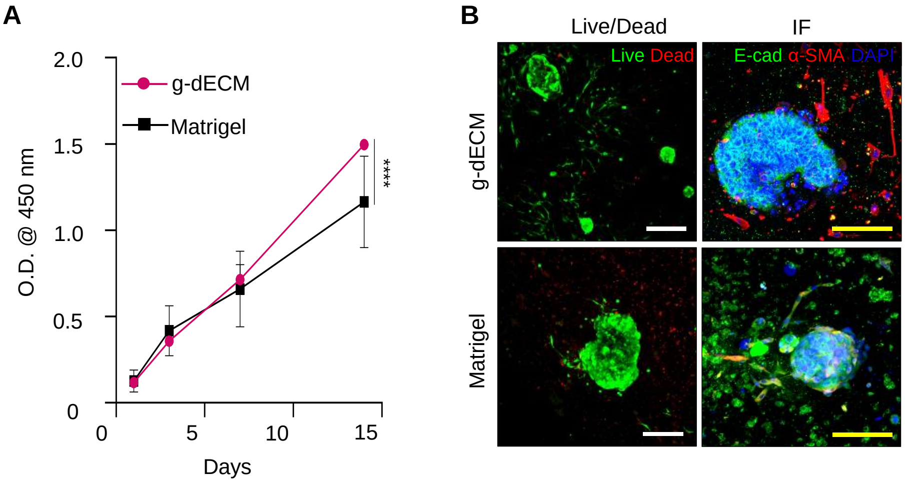

Figure S2. Assessment of pGC tissue characteristics based on material composition. (A) Comparison of proliferation rates of pGC tissues encapsulated in g-dECM and Matrigel for 14 days. Data represent mean ± S.D.(n=3, **** $p < 0.0001$). (B) Live/dead staining and immunofluorescence staining images of E-cadherin (green), α-SMA (red), and DAPI (blue) in pGC tissues encapsulated in g-dECM and Matrigel at day 7, respectively. White scale bar: 250 µm; Yellow scale bar: 100 µm

Figure S3.

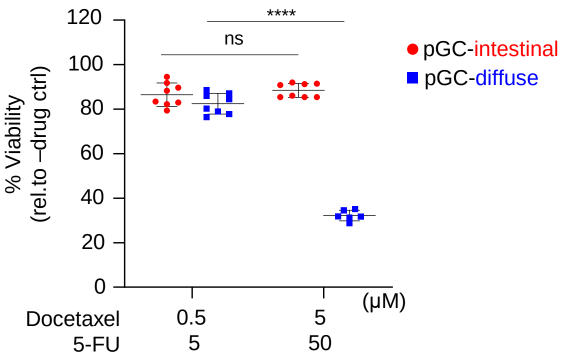

Figure S3. Evaluation of the efficacy of Docetaxel and 5-FU combination regimens in pGC model. Two concentration combinations for Docetaxel and 5-FU combination therapy. Data represent mean ± S.D. (n=3, ****p< 0.0001, ns; not significant)and analysed using the ordinary two-way ANOVA with Tukey's multiple comparisons test including p-values.

Figure S4.

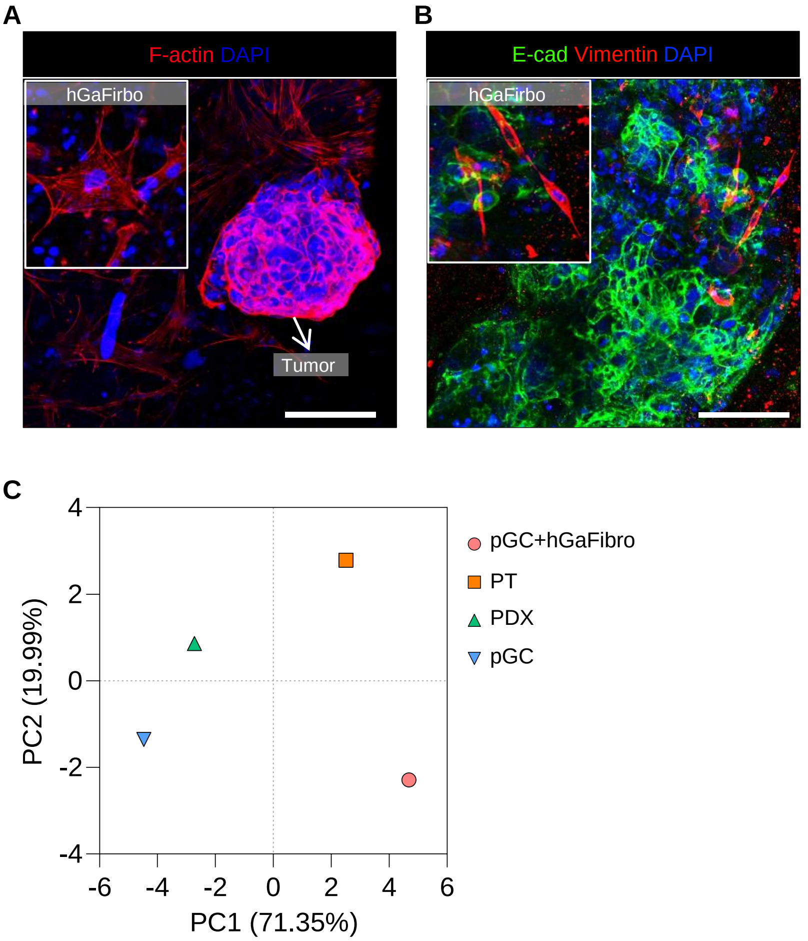

Figure S4. Observation of close crosstalk between patient-derived gastric cancer cells and hGaFibro in pGC with hGaFibro model. (A) Immunofluorescence staining images of F-actin (red) and DAPI (blue), and (B) E-cad (green), Vimentin (red), and DAPI (blue) in pGC-intestinal tissues cultured in g-dECM at day 7. Scale bar: 100 µm. (C) 2D Principal component analysis plot of the expression of EMT-related genes according to similarity of 4 different groups.

Figure S5.

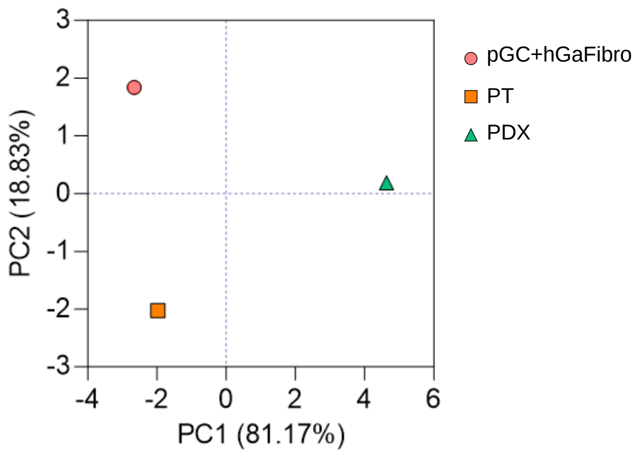

Figure S5. 2D Principal component analysis plot of the expression of 20 drug resistance-related genes in 3 different groups.

Figure S6.

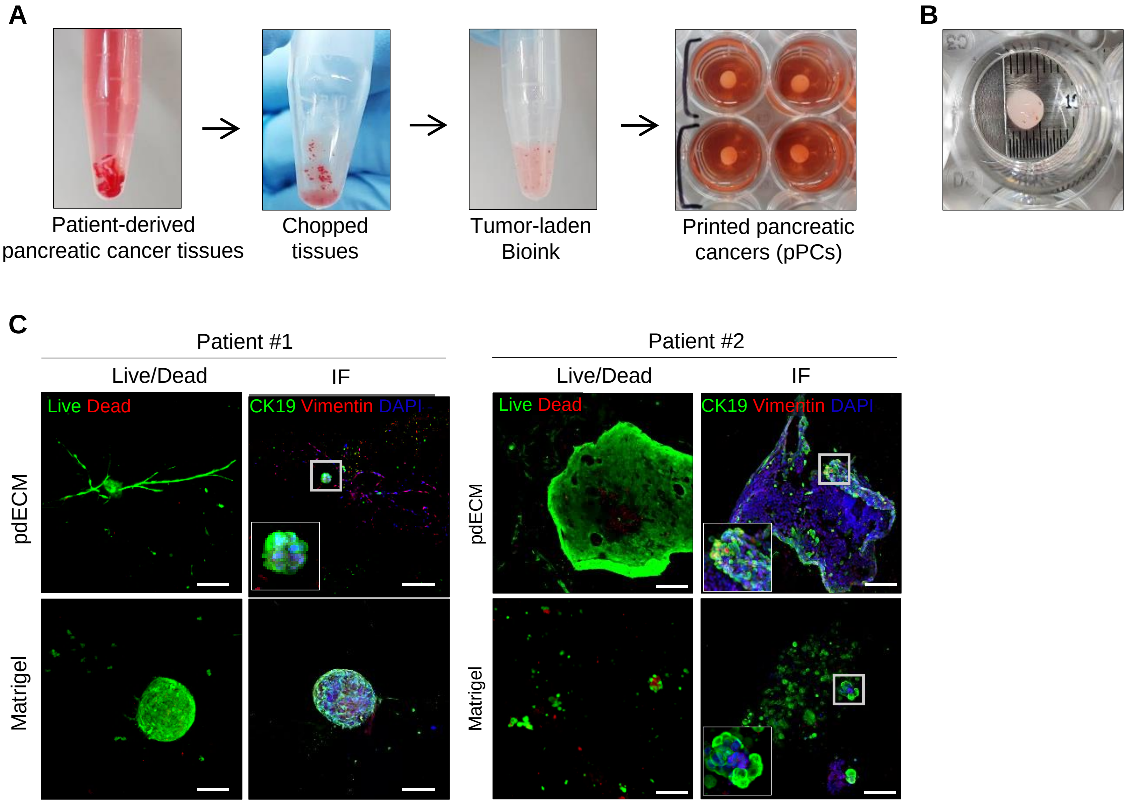

Figure S6. Verification of the possibility of expansion to various solid cancer models by producing a printed pancreatic cancer model. (A) Summary of the printed pancreatic cancer (pPC) model generation process. (B) Confirmation of pPC size. (C) Live/dead stanning and immunofluorescence staining images of cytokeratin 19 (CK19, green), Vimentin (red), and DAPI (blue) in pPC tissues in g-dECM and Matrigel at days 14. Scale bar: 200 μm.

Figure S7.

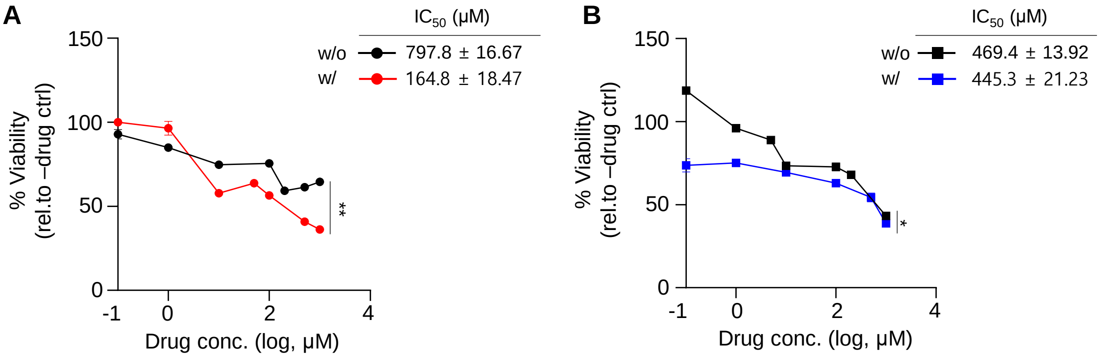

Figure S7. Identification of 5-FU response-related features in pGC with helper T cells model (A) Dose-response curves after 3 days of treatment with 5-FU under various conditions of pGC-intestinal tissue with the presence (red) and absence (black) of helper T cells. (B) Dose-response curves after 3 days of treatment with 5-FU under various conditions of pGC-diffuse tissue with the presence (blue) and absence (black) of helper T cells. Data represent mean ± S.D. (n=3, *$p < 0.05$, **$p < 0.01$) and analysed using paired t-test including *p*-values.

Figure S8.

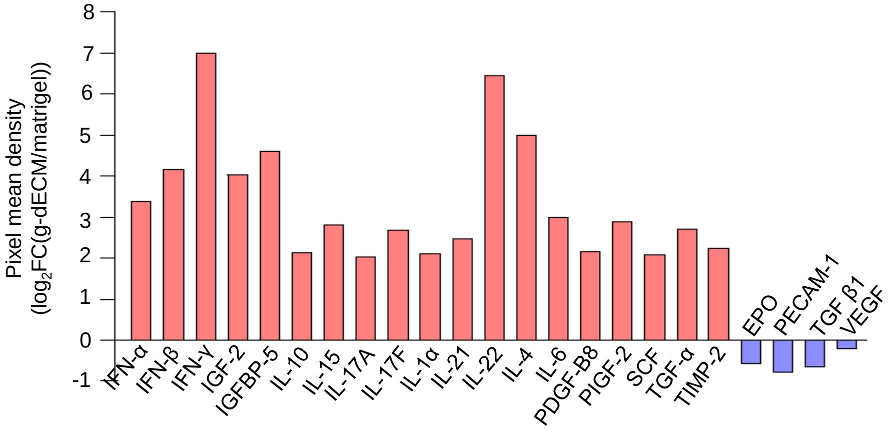

Figure S8. Investigation of residual cytokine content in g-dECM compared to Matrigel.
